# 2D:4D Asymmetry and Gender Differences in Academic Performance

**DOI:** 10.1371/journal.pone.0046319

**Published:** 2012-10-08

**Authors:** John V. C. Nye, Gregory Androuschak, Desirée Desierto, Garett Jones, Maria Yudkevich

**Affiliations:** 1 Department of Economics, George Mason University, Fairfax, Virginia, United States of America; 2 Laboratory for Institutional Analysis of Economic Reforms, National Research University “Higher School of Economics”, Moscow, Russia; 3 University of the Philippines School of Economics, Quezon City, Philippines; Baylor College of Medicine, United States of America

## Abstract

Exposure to prenatal androgens affects both future behavior and life choices. However, there is still relatively limited evidence on its effects on academic performance. Moreover, the predicted effect of exposure to prenatal testosterone (T)–which is inversely correlated with the relative length of the second to fourth finger lengths (2D:4D)–would seem to have ambiguous effects on academic achievement since traits like aggressiveness or risk-taking are not uniformly positive for success in school. We provide the first evidence of a non-linear, quadratic, relationship between 2D:4D and academic achievement using samples from Moscow and Manila. We also find that there is a gender differentiated link between various measures of academic achievement and measured digit ratios. These effects are different depending on the field of study, choice of achievement measure, and use of the right hand or left digit ratios. The results seem to be asymmetric between Moscow and Manila where the right (left) hand generates inverted-U (U-shaped) curves in Moscow while the pattern for hands reverses in Manila. Drawing from unusually large and detailed samples of university students in two countries not studied in the digit literature, our work is the first to have a large cross country comparison that includes two groups with very different ethnic compositions.

## Introduction

Performance in schooling is known to be dependent on cognitive ability, family background, and social status, but it is also heavily influenced by biological and psychological traits independent of or even orthogonal to standard notions of cognitive ability. These include aggressiveness or self-confidence, conscientiousness, and/or willingness to take risks. (For cognitive ability, see [1]. For noncognitive skills, see [2] and [3]).

Some of these characteristics may derive partly from prenatal exposure to androgenic steroids. The most common marker for measuring prenatal androgens is the second-to-fourth finger digit length ratio (henceforth 2D:4D) with relatively longer fourth fingers (lower 2D:4D) indicating higher fetal androgens [4]. Previous work has shown links between digit ratios and success in competitive sports, preference for risk, and success in high frequency financial trading (e.g. [5]–[6] for sports; [7]–[9] for risk; and [10] on financial trading).

However, the most recent large surveys do not support robust, within-sex correlations between 2D:4D and the masculinity/femininity personality dimensions [11] and only small effects for 2D:4D and aggression [12]. What seems to persist are the links to sporting ability and to risk taking and financial trading mentioned above.

Ties to academic achievement are even less well-explored. There is some limited work on the relationship between 2D:4D and academic performance but the findings are mixed and often based on limited samples. Romano found that adult males’ 2D:4D ratios positively predicted examination grades while females’ marks were uncorrelated with these ratios [13]. Others have studied British school children’s digit ratios and their correlations with their numeracy and literacy [14]. Digit ratios were not found to be significant for the group as a whole but there were sex based differences whereby lower digit ratios predicted numeracy for boys and higher digit ratios predicted higher literary SAT scores for girls – though in both cases the effects were small. Similarly, Bull et al. [15] found no correlations between the digit ratios and numerical or visual-spatial tasksof children.

Brosnan et al. [16] considered a small group of computer science students to see if prenatal testosterone exposure was related to performance and computeranxiety, however they found few correlations and no sex-related differences in grades. However, lower computer anxiety was associated with lower 2D:4D ratios.

The strongest claims on 2D:4D effects that might be relevant for understanding academic achievement are to be found in Branas-Garza and Rustichini’s [17] work which follows up on the demonstrated link between prenatal T exposure (low 2D:4D) and success in financial trading. They find links between low measured 2D:4D and higher performance on tests of abstract reasoning, as well as risk taking. This work might suggest that we should observe low 2D:4D predicting higher academic achievement to the extent that the abstract reasoning relationship is dominant. However, as Branas-Garza and Rustichini [17] note, the interaction between the gender specific effects of 2D:4D and its links to abstract reasoning and risk taking are fairly complex especially considering the much stronger links between abstract reasoning and risk-taking for males. There seems to be no link between digit ratio and risk taking for females.

This work, seen in light of the earlier diverse findings showing at best weak links between 2D:4D and academic performance suggests that there might be strongly sex differentiated effects; further, the unreliable findings across studies could be driven by nonlinearity in the relationship between testosterone and later outcomes. Some characteristics associated with high testosterone could plausibly have non-linear effects on performance – some risk-taking or aggressiveness, for instance, might be beneficial, but too much might lead to destructive behavior (e.g. [18] shows low digit ratios correlated with increased tendency to alcohol dependency). Also, the importance of abstract reasoning in determining achievement might vary by field of study and program.

Sapienza et al. [8] were among the first to highlight potential nonlinearities as confounding the effects of prenatal testosterone exposure that might result in insignificant linear estimations but did not directly test for non-linear effects themselves.

## Materials and Methods

The various findings from the literature suggest that the effect of 2D:4D on academic outcomes may be more complex than a linear relationship. There may be many other relevant factors that affect academic outcomes that happen to be correlated with 2D:4D, and any effect of 2D:4D on academic outcomes obtained from a simple linear specification may be an incomplete approximation.

To verify whether the effect could actually be non-linear, and would vary across academic fields and between genders, we specify the following model:

(1)where the academic outcome *W* of individual *i* of gender in field *f* with digit ratio *DR* of hand is a quadratic function of *DR*. Note that equation (1) not only controls for gender and academic field, but also allows for the possibility that results may differ depending on which hand is used. This is because the physiology of how and to what extent prenatal testosterone is manifested in digit ratios is still unclear, which makes it difficult to ascertain whether it is the left or the right hand that best reflects prenatal testosterone. For example, [6] and [16] get significant results with both hands’ digit ratios, but [5] gets consistency for both hands only for males and only left hand results for females. Articles [9], [10], [13] and [17] only get significance for males’ right hands, while [8], [14], and [15] use averages of both hands.

To empirically test equation (1), we use two different cross-sectional datasets – one is a sample of over 700 students from the Higher School of Economics (HSE) in Moscow, and the other is a sample of about 120 students from the University of the Philippines School of Economics (UPSE) in Manila. For both Moscow and Manila, all the students in the samples were recruited for the study in a manner consistent with local protocols for human subject research. Though no signed consent forms were obtained, permission for the study was formally obtained at the HSE and the UPSE in accordance with local practice. In addition, the overall survey and research design was reviewed by the George Mason University Office of Research Subjects Protection and it was determined that no review by the Human Subjects Review Board was necessary for participation by the two authors representing GMU who were not directly involved in collecting the survey information presented to them in anonymous form.

In Moscow, measurements of the second and fourth fingers of both the left and right hands of all the subjects were taken by two research assistants using a laser caliper (with the exception of those subjects who had stated in the questionnaire that they had broken their second and/or fourth finger – these were then omitted). In Manila, we had the subjects photocopy their left and right hands, and from these, two research assistants obtained the lengths of the second and fourth fingers using tape measures. Whether by laser caliper or tape measure, finger length is measured as the distance between the middle of the line at the base of the finger up to the point on the fingertip that is perpendicular to that base. Note that in both Moscow and Manila, subjects were allocated among the research assistants, but each assistant measured both the left and right-hand fingers of the subjects assigned to her. Thus, while there may be some variability in the measurements across subjects, we do not expect any biased difference between the measurements of the left hand and the right hand and/or between the measurements of the second and fourth fingers of each hand.

From these measurements, each subject’s digit ratio was computed by dividing the length of the subject’s second finger to the length of her fourth finger, for her left and right hands. For both Moscow and Manila samples, we thus have two proxies for , denoted as Left hand 2D:4D and Right hand 2D:4D.

We use several proxies for individual academic outcomes . For the Moscow sample, we have information on test scores on the college entrance exam–the Unified State Exam (USE)–particularly the Math Score and the Russian (language) Score. (It should be noted that there was an old version of the USE which was in a different form and used a different grading scale. This old USE was taken by the oldest students in the original sample and only as an option, unlike the new version of the USE which is compulsory. To get a consistent set of students for the final sample, we only included the younger students, i.e. those who took the new version of the USE. However, as a robustness check, we also ran regressions using the original sample in which students who took the old USE were included, after re-scaling their scores to approximate the new USE. The results are generally similar to the ones reported in this paper and can be provided upon demand).

We also have data on whether the subject was admitted to HSE based on high scores in pre-college competitions called Olympiads; whether the subject was a recipient of high school honors; and whether the subject was admitted to HSE with a full academic scholarship (virtually all HSE scholarships are based on academic criteria only using non-subjective formulae). For these we constructed the corresponding binary variables Olympiad, High School Honors and Full Scholarship.

For the Manila sample, the subjects provided their grades for all economics courses taken to date and their grades for all mathematics courses taken to date. We converted these to the US grading scale (using the official guidelines of the University of the Philippines) and computed the Economics Weighted Average and the Mathematics Weighted Average according to the University’s convention of using the number of units of the course as its weight.

We also have data on the subjects’ gender from both Moscow and Manila. In addition, because HSE is further divided into different faculties, we create binary variables indicating the particular Faculty to which each Moscow subject belongs: Faculty(Economics), Faculty(Law), Faculty(Management) and Faculty(Political Science).

## Results

### Summary Statistics and Bivariate Correlations

Tables 1 and 2 list all the variables used in this study and provides some descriptive statistics. Note that for the full sample, and separately among females and among males, the mean Math Score is lower than mean Russian Score, and the mean Mathematics Weighted Average is lower than the Economics Weighted Average. In the Manila sample, females on average have significantly lower Economics and Mathematics Weighted Averages than males, while in Moscow, females on average have significantly higher Russian Scores than males, and that they are also more likely to have High School Honors and Full Scholarship. Furthermore, the choice of Faculty may also be gender-differentiated, with females in Moscow significantly more likely to be in Political Science but less likely to be in Economics.

**Table 1:** Descriptive Statistics for the Full Sample and by Gender – Manila.

*Manila*					
Variables	N	Mean	Std. Dev.	Min	Max
Outcome Variables					
Economics Weighted Average (US Score Equivalent)					
Full sample	121	2.542	0.672	1.250	4
Females***	72	2.411	0.663	1.250	4
Males***	49	2.734	0.645	1.614	3.792
Mathematics Weighted Average (US Score Equivalent)					
Full sample	123	2.425	0.817	0.491	4
Females**	74	2.285	0.754	0.491	4
Males**	49	2.635	0.871	1	4
Explanatory Variables					
Right hand 2D:4D					
Full sample	123	0.986	0.038	0.878	1.129
Females***	74	0.996	0.037	0.922	1.129
Males***	49	0.971	0.034	0.878	1.043
Left hand 2D:4D					
Full sample	123	0.964	0.033	0.859	1.060
Females**	74	0.969	0.034	0.859	1.060
Males**	49	0.956	0.030	0.877	1.028
Female	123	0.602	0.492	0	1
Male	123	0.398	0.492	0	1

Note: *** 1%, ** 5% Significant difference between the mean values for the female and male subsamples

**Table 2:** Descriptive Statistics for the Full Sample and by Gender – Moscow.

*Moscow*					
Variables	N	Mean	Std. Dev.	Min	Max
Outcome Variables					
Math Score (Unified State Exam)					
Full sample	277	70.484	12.292	30	100
Females	152	70.559	11.570	44	95
Males	125	70.392	13.163	30	100
Russian Score (Unified State Exam)					
Full sample	421	78.948	9.287	54	100
Females***	242	81.277	8.714	60	100
Males***	179	75.799	9.129	54	100
Olympiad					
Full sample	770	0.300	0.459	0	1
Females	446	0.296	0.457	0	1
Males	323	0.307	0.462	0	1
High School Honors					
Full sample	755	0.404	0.491	0	1
Females***	435	0.492	0.501	0	1
Males***	319	0.285	0.452	0	1
Full Scholarship					
Full sample	770	0.704	0.457	0	1
Females	447	0.747	0.435	0	1
Males	322	0.643	0.480	0	1
Explanatory Variables					
Faculty (Economics)					
Full sample	796	0.273	0.446	0	1
Females	449	0.245	0.431	0	1
Males	327	0.306	0.461	0	1
Faculty (Law)					
Full sample	796	0.319	0.466	0	1
Females	449	0.318	0.466	0	1
Males	327	0.330	0.471	0	1
Faculty (Management)					
Full sample	796	0.205	0.404	0	1
Females	449	0.212	0.409	0	1
Males	327	0.208	0.406	0	1
Faculty (Political Science)					
Full sample	796	0.204	0.403	0	1
Females***	449	0.225	0.418	0	1
Males***	327	0.156	0.363	0	1
Right hand 2D:4D					
Full sample	814	0.989	0.037	0.857	1.134
Females***	449	0.994	0.038	0.857	1.134
Males***	327	0.983	0.033	0.902	1.082
Left hand 2D:4D					
Full sample	814	0.990	0.034	0.876	1.115
Females***	449	0.995	0.035	0.890	1.115
Males***	327	0.984	0.033	0.876	1.072
Female	776	0.579	0.494	0	1
Male	776	0.421	0.494	0	1

Note: *** 1%, ** 5% Significant difference between the mean values for the female and male subsamples

For both Manila and Moscow, the mean values of the Right hand 2D:4D and Left hand 2D:4D are significantly different for males and females, with females having significantly higher Right hand 2D:4D and Left hand 2D:4D than males. This suggests that, on average, females have significantly less prenatal testosterone exposure than males. In addition, the mean values of Right 2D:4D for females are similar across Manila and Moscow, but the mean Left 2D:4D is lower for females in Manila than in Moscow. Judging only by the left hand, this suggests that female Manila students may have more prenatal testosterone on average than female Moscow students. Male Manila students also may have higher prenatal testosterone than male Moscow students, as the former’s mean Right and Left 2D:4D are lower than the latter’s.

Tables 3, 4, 5, and 6 present bivariate correlations among all the variables for the full sample, and separately for the female and male subsamples. Note that the different academic outcome variables are significantly and positively correlated, with the exception of the Olympiad variable in Moscow which is negatively correlated with Math scores and Russian scores, but positively correlated with High School Honors and Full Scholarship. The correlations, however, are less than half, even between the Mathematics and Economics Weighted Averages in Manila, or between Math and Russian Scores in Moscow. (That the former correlation is larger than the latter may be expected, since economics courses are mathematics-based.)

**Table 3:** Bivariate Correlations for the Full Sample – Manila.

*Manila (Full Sample)*					
*Variables*	Economics Weighted Average (US score equivalent)	Mathematics Weighted Average (US score equivalent)	Right hand 2D:4D	Left hand 2D:4D	Female
Economics Weighted Average (US score equivalent)	1.000				
Mathematics Weighted Average (US score equivalent)	0.474*	1.000			
Right hand 2D:4D	0.069	0.055	1.000		
Left hand 2D:4D	0.042	0.061	0.554*	1.000	
Female	−0.237*	−0.211*	0.327*	0.205*	1.000

Note: * Significant at 10%, **5%, ***1%

**Table 4:** Bivariate Correlations By Gender – Manila.

*Manila (By Gender)*								
*Variables*	Economics Weighted Average (US score equivalent)		Mathematics Weighted Average (US score equivalent)		Right hand 2D:4D		Left hand 2D:4D	
	Female	Male	Female	Male	Female	Male	Female	Male
Economics Weighted Average (US score equivalent)	1.000	1.000						
Mathematics Weighted Average (US score equivalent)	0.429*	0.472*	1.000	1.000				
Right hand 2D:4D	0.149	0.182	0.215***	0.019	1.000	1.000		
Left hand 2D:4D	0.059	0.145	0.048	0.202	0.559*	0.466*	1.000	1.000

Note: * Significant at 10%, **5%, ***1%

**Table 5:** Bivariate Correlations for the Full Sample – Moscow.

*Moscow (Full Sample)*												
*Variables*	Math Score	Russian Score	Olympiad	High School Honors	Full Scholarship	Faculty (Economics)	Faculty (Law)	Faculty (Management)	Faculty (Political Science)	Right hand 2D:4D	Left hand 2D:4D	Female
Math Score	1.000											
Russian Score	0.404*	1.000										
Olympiad	−0.176*	−0.076	1.000									
Honors	0.299*	0.366*	0.107*	1.000								
Full Scholarship	0.096	0.335*	0.418*	0.261*	1.000							
Faculty (Economics)	0.588*	0.203*	0.235*	0.165*	0.014	1.000						
Faculty (Law)	−0.396*	−0.103*	−0.099*	−0.033	0.091*	−0.419*	1.000					
Faculty (Management)	−0.145*	−0.121*	−0.150*	−0.129*	−0.241*	−0.311*	−0.347*	1.000				
Faculty (Political Science)	−0.369*	−0.009	0.005	−0.014	0.122*	−0.310*	−0.346*	−0.257*	1.000			
Right hand 2D:4D	0.005	0.022	0.015	0.053	0.070*	−0.054	0.009	0.117*	−0.068*	1.000		
Left hand 2D:4D	−0.059	0.077	0.019	0.051	0.064*	−0.008	−0.024	0.067*	−0.032	0.564*	1.000	
Female	0.007	0.292*	−0.011	0.208*	0.113*	−0.068*	−0.012	0.004	0.086*	0.159*	0.156*	1.000

Note: * Significant at 10%, **5%, ***1%

**Table 6:** Bivariate Correlations By Gender – Moscow.

*Moscow (By Gender)*												
*Variables*	Math Score		Russian Score		Olympiad		High School Honors		Full Scholarship			
	Female	Male	Female	Male	Female	Male	Female	Male	Female	Male		
Math Score	1.000	1.000										
Russian Score	0.448*	0.388*	1.000	1.000								
Olympiad	−0.188**	−0.165	−0.129**	−0.001	1.000	1.000						
Honors	0.372*	0.239*	0.317*	0.336*	0.082***	0.151*	1.000	1.000				
Full Scholarship	0.127	0.066	0.304*	0.308*	0.366*	0.493*	0.182*	0.330*	1.000	1.000		
Faculty (Economics)	0.623*	0.553*	0.252*	0.191**	0.199*	0.281*	0.219*	0.142**	−0.014	0.066		
Faculty (Law)	−0.410*	−0.383*	−0.064	−0.131	−0.077	−0.126**	−0.053	0.005	0.079***	0.108		
Faculty (Management)	−0.172**	−0.117	−0.192*	−0.024	−0.127*	−0.183*	−0.116**	−0.165*	−0.235*	−0.255*		
Faculty (Political Science)	−0.362*	−0.382*	−0.037	−0.064	0.005	0.007	−0.051	−0.005	0.154*	0.057		
Right hand 2D:4D	−0.046	0.062	−0.037	−0.045	0.006	0.036	0.069	−0.061	0.011	0.114**		
Left hand 2D:4D	−0.093	−0.027	−0.024	0.093	0.027	0.013	0.026	0.008	−0.010	0.122**		
**Variables**	**Faculty (Economics)**		**Faculty (Law)**		**Faculty (Management)**		**Faculty (Political Science)**		**Right hand 2D:4D**		**Left hand 2D:4D**	
	**Female**	**Male**	**Female**	**Male**	**Female**	**Male**	**Female**	**Male**	**Female**	**Male**	**Female**	**Male**
Faculty (Economics)	1.000	1.000										
Faculty (Law)	−0.389*	−0.466*	1.000	1.000								
Faculty (Management)	−0.295*	−0.340*	−0.354*	−0.360*	1.000	1.000						
Faculty (Political Science)	−0.307*	−0.285*	−0.368*	−0.302*	−0.279*	−0.220*	1.000	1.000				
Right hand 2D:4D	−0.033	−0.085	−0.029	0.081	0.147*	0.071	−0.079***	−0.077	1.000	1.000		
Left hand 2D:4D	0.024	−0.041	−0.067	0.049	0.115**	−0.007	−0.063	−0.004	0.574*	0.542*	1.000	1.000

Note: * Significant at 10%, **5%, ***1%

Note also that while Left hand 2D:4D and Right hand 2D:4D are positively correlated in both samples, the correlation is not very high – only 0.55 for Manila and 0.56 for Moscow when aggregating females and males. The correlations appear larger for females than males, with 0.56 for females in Manila and 0.47 for males, and 0.57 for females in Moscow and 0.52 for males. This indicates that prenatal testosterone may be expressed differently between the hands, and between females and males, and suggests that regression results may differ significantly by gender and depending on which hand is used. In fact, Full Scholarship is significantly positively correlated with both Left and Right 2D:4D for males in Moscow, while the Mathematics Weighted Average is correlated with Right 2D:4D for females in Manila.

Gender also appears to have a direct correlation with academic outcomes and digit ratios. In Manila, being female is negatively correlated with the Economics and Mathematics Weighted Average, and positively correlated with Left and Right 2D:4D. Being female is also positively correlated with digit ratios in Moscow, but unlike Manila, it is positively correlated with academic outcome variables, specifically, Russian Score, High School Honors and Full Scholarship.

Lastly, note that the Faculty variables in Moscow are significantly correlated with the academic outcome variables for the full sample, and when subdividing by gender. In particular, Faculty (Economics) is positively related to all outcomes except Full Scholarship, while Faculty (Law), Faculty (Management), and Faculty (Political Science) are negatively related to most of the outcome variables.

### Non-linear (Quadratic) Association between 2D:4D and Academic Outcomes

**Figure 1:**
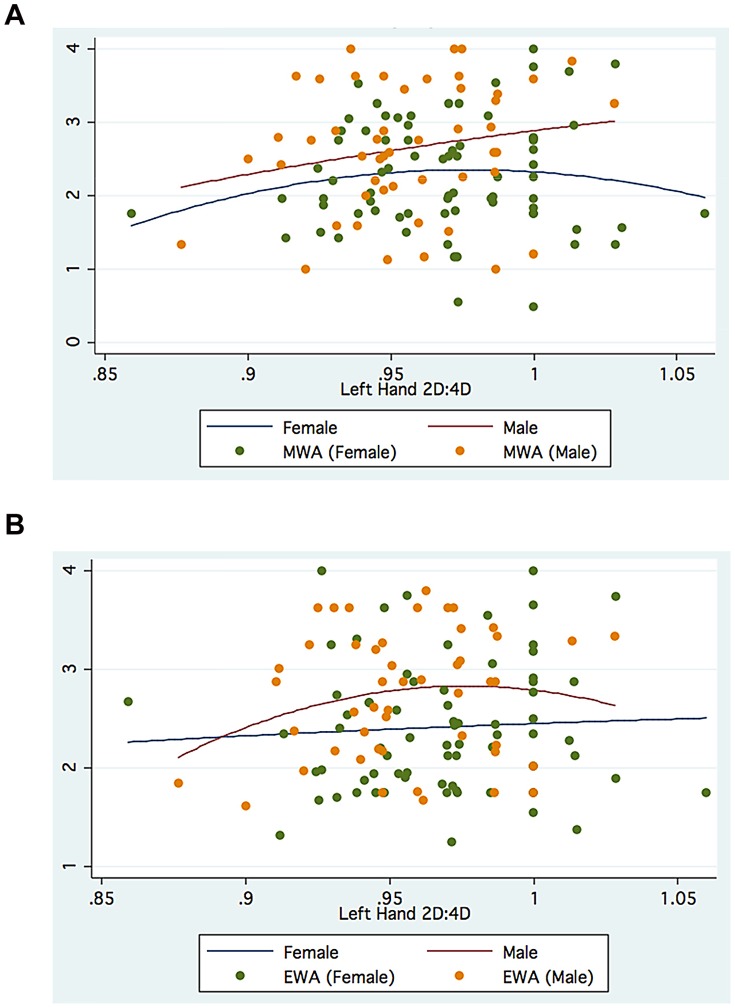
a. Left 2D:4D and Mathematics Weighted Average (MWA): Manila. b. Left 2D:4D and Economics Weighted Average (EWA): Manila.

**Figure 2:**
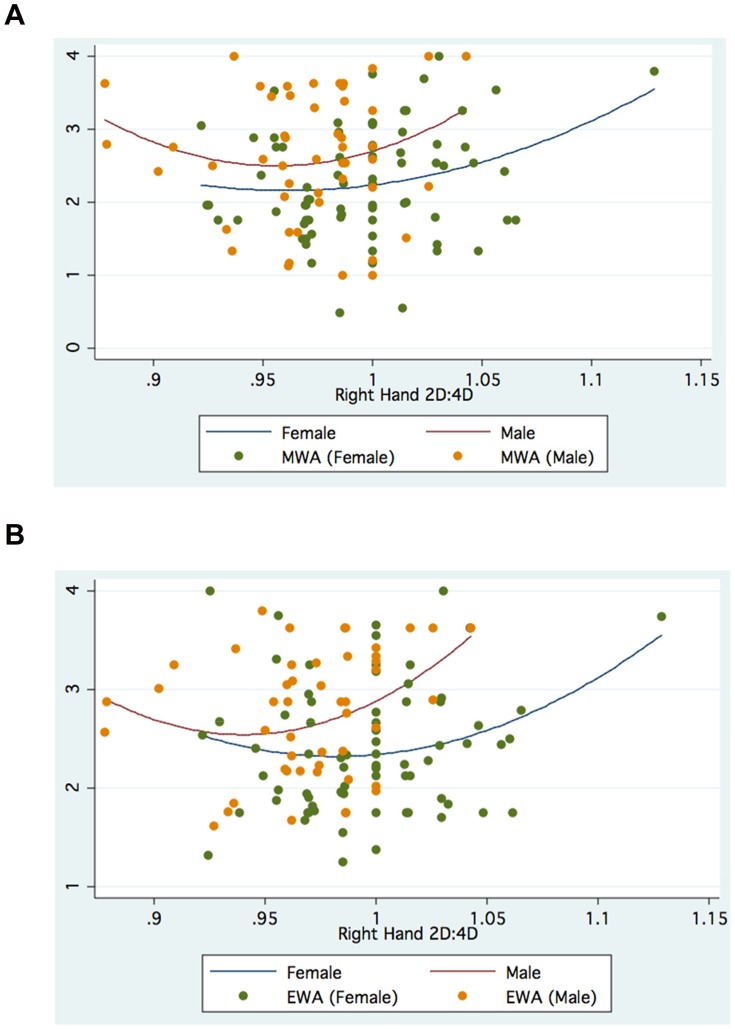
a. Right 2D:4D and Mathematics Weighted Average (MWA): Manila. b. Right 2D:4D and Economics Weighted Average (EWA): Manila.

**Figure 3:**
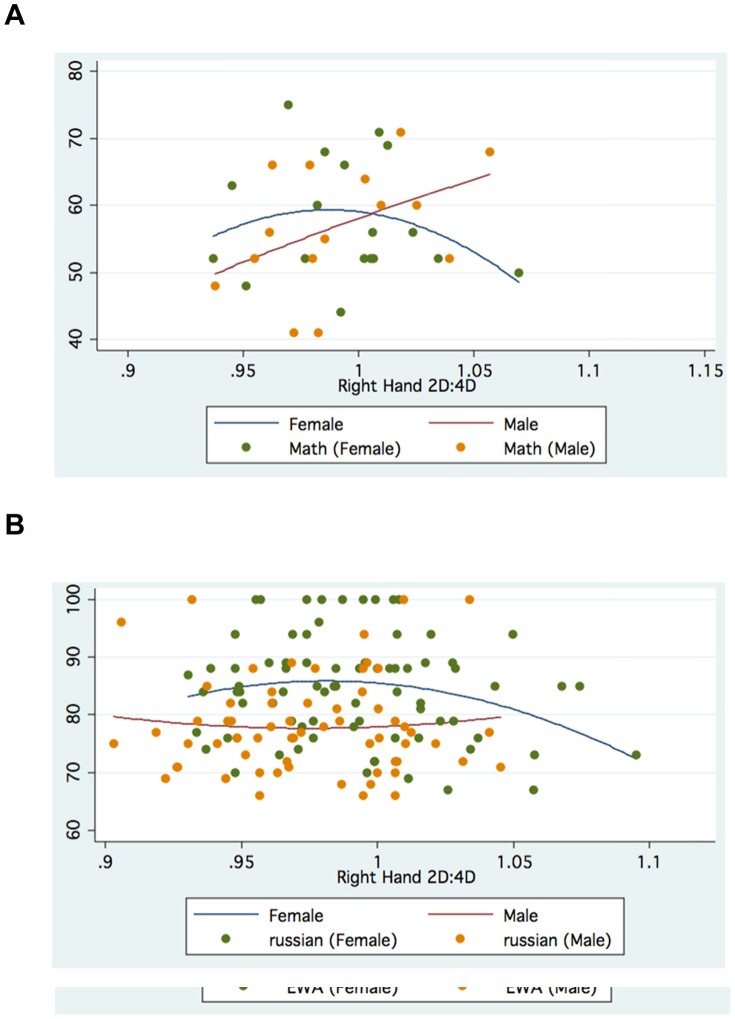
a. Right 2D:4D and Math Scores: Moscow Faculty(Law). b. Right 2D:4D and Russian Scores: Moscow Faculty(Economics).

**Figure 4:**
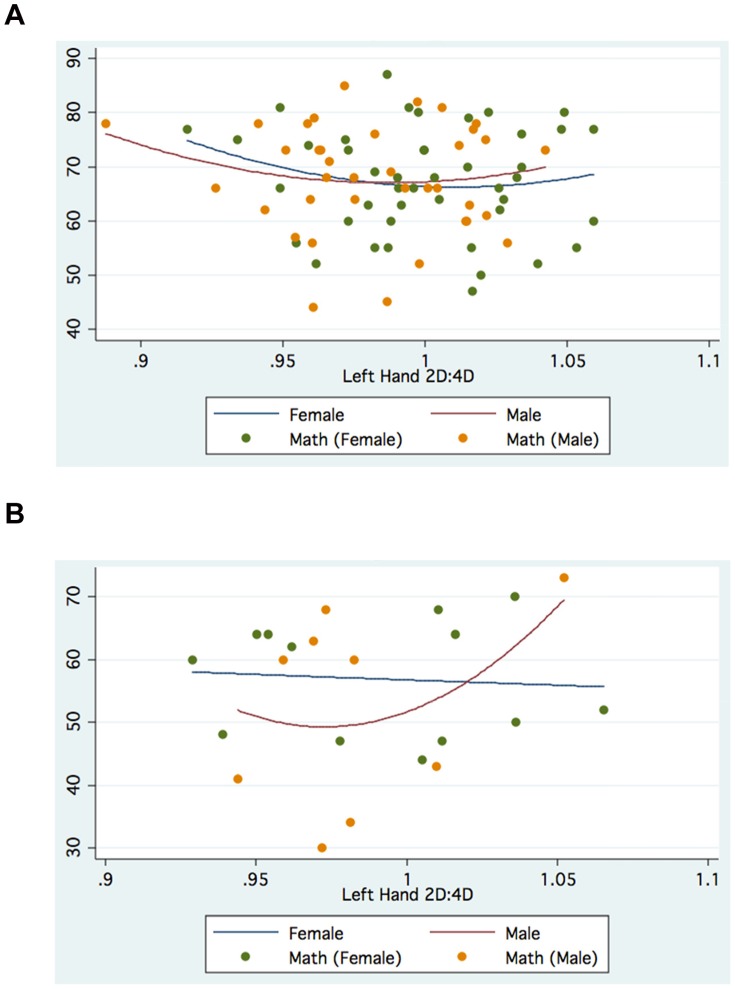
a. Left 2D:4D and Math Scores: Moscow Faculty(Management). b. Left 2D:4D and Russian Scores: Moscow Faculty(Political Science).

The foregoing suggests that gender, choice of Faculty and hand measured can modify the association between digit ratio and academic outcomes. Figures 1a, 1b, 2a, 2b, 3a, 3b, 4a, and 4b further suggest that such associations may be nonlinear, specifically quadratic, for both Manila and Moscow.

The Manila graphs depict an inverted-U relationship between Mathematics Weighted Average and Left 2D:4D for females (but not so for males), as well as an inverted-U relationship between Economics Weighted Average and Left 2D:4D for males (but not for females). Note, however, that when the Right 2D:4D is used, the non-linear relationships are now U-shaped for both males and females.

On the other hand, the Moscow graphs generally show inverted-U relationships between various academic outcomes and Right 2D:4D, even within different Faculties (especially for females), while the relationships between outcomes and Left 2D:4D are mostly U-shaped (especially for males).

### Regression Analysis

We now provide more rigorous regression-based tests of our hypothesis that digit ratios affect academic outcomes in a quadratic manner and that the effects are differentiated between genders, Faculty types, and left and right hands.

The next tables report the results from OLS regressions of equation (1) for the continuous dependent variables in Moscow and Manila, i.e. Math Scores, Russian, Scores, Economics Weighted Average and Mathematics Weighted Average. For the binary dependent variables High School Honors, Olympiad and Full Scholarship, equation (1) is interpreted as a Linear Probability Model (LPM). As an alternative to the LPM, logit regressions are also reported. Whenever the OLS/LPM results show that there is a significant quadratic relationship between 2D:4D and the academic outcome, we also compute the optimal value of 2D:4D that maximizes or minimizes this academic outcome. That is, maxima are computed for significant inverted-U relationships, while minima are computed for significant U-shaped relationships. Note that an inverted-U (U-shaped) relationship is implied by a positive (negative) estimated coefficient *b*_1_ and a negative (positive) estimated coefficient *b*_2_. (The maxima/minima are not computed for the logit regressions since unlike in the OLS/LPM, the marginal effect of the digit ratio is not readily computed from the estimated coefficients in the logit regressions. Although the marginal effects from logit regressions are not reported here, they are very similar to the LPM, and can be provided by the authors upon request. However, to get an approximate comparison between LPM and Logit results, one can divide the estimated logit coefficients by 4). Lastly, we also indicate whether the variables are significant in backward stepwise regressions, specifically those whose p-values in backward stepwise regressions are equal to or less than the chosen tolerance level p = 0.10 and thus ought not to be removed from the model.

Tables 7, 8, 9, and 10 present the various regression results for Moscow when Left 2D:4D is used. The results for subsamples Female and Male are reported in separate columns. However, the results for the various Faculty subsamples are concisely reported in single specifications involving interaction terms of the various Faculty binary variables with 2D:4D, and with the square of 2D:4D. Thus, columns with no interaction terms are the results from regressions by gender only, while those with interaction terms are results from regressions by gender and faculty type. To interpret the latter columns, note that the chosen base group is Faculty(Economics), such that the coefficients from the uninteracted 2D:4D and square of 2D:4D pertain to the estimated coefficients *b*_1_ and *b*_2_ for subsample Faculty (Economics) (that is, when all Faculty dummies are zero). For subsample Faculty (Law), that is, when Faculty(Law) is one (and other Faculty dummies are zero), its coefficient *b*_1_ is thus equal to the sum of the coefficients of 2D:4D and of 2D:4D × Faculty(Law), while the coefficient *b*_2_ is the sum of the coefficients of square of 2D:4D and of square of 2D:4D × Faculty(Law).

**Table 7:** OLS Regressions of Russian Score and Math Score on Left Hand 2D:4D and its Square.

*Moscow*								
	Russian Score				Math Score			
	Female		Male		Female		Male	
Explanatory Variables	OLS	OLS	OLS	OLS	OLS	OLS	OLS	OLS
Left Hand 2D:4D	−752.148	−557.914 +	−713.831	−857.401	1455.801	641.322	−1680.256** +	−498.408
	*(734.042)*	*(622.633)*	*(641.754)*	*(699.541)*	*(1218.6705)*	*(809.7495)*	*(723.913)*	*(610.858)*
Square of Left Hand 2D:4D	374.309	257.180	374.938	453.383	−744.247	−335.559	849.140** +	267.912
	*(366.889)*	*(313.841)*	*(327.8416)*	*(362.109)*	*(607.0971)*	*(405.1691)*	*(371.962)*	*(309.741)*
Left Hand 2D:4D × Faculty (Law)		−77.678** +		20.373		−33.9398 +		40.912
		*(34.992)*		*(46.364)*		*(49.2521)*		*(57.116)*
Left Hand 2D:4D × Faculty (Management)		16.970		−57.745		−21.6169 +		26.279
		*(44.868)*		*(59.687)*		*(52.6171)*		*(49.523)*
Left Hand 2D:4D × Faculty (Political Science)		−115.271** +		45.408		−49.9604 +		−186.639* +
		*(45.557)*		*(116.259)*		*(54.1926)*		*(104.135)*
Square of Left Hand 2D:4D × Faculty (Law)		73.579** +		−25.363		14.244		−62.442 +
		*(34.958)*		*(47.100)*		*(49.0035)*		*(56.639)*
Square of Left Hand 2D:4D × Faculty (Management)		−23.475 +		55.889		11.500		−36.531 +
		*(44.660)*		*(61.280)*		*(52.5432)*		*(50.337)*
Square of Left Hand 2D:4D × Faculty (Political Science)		112.109** +		−49.721		29.306		163.886 +
		*(45.878)*		*(119.275)*		*(54.7281)*		*(104.732)*
Constant	458.627	384.719	414.762	482.390	−640.098	−227.823	900.529**	308.294
	*(366.954)*	*(308.834)*	*(313.970)*	*(337.958)*	*(611.1653)*	*(404.5169)*	*(351.582)*	*(300.450)*
*R-squared (OLS)*	*0.007*	*0.128*	*0.014*	*0.070*	*0.026*	*0.491*	*0.0151*	*0.4415*
*Adjusted R-squared (OLS)*	−*0.001*	*0.098*	*0.003*	*0.026*	*0.013*	*0.463*	−*0.001*	*0.403*
*R-squared (Stepwise)*	*0.000*	*0.125*	*0.000*	*0.000*	*0.000*	*0.484*	*0.0151*	*0.437*
*N*	*242*	*242*	*179*	*179*	*152.000*	*152.000*	*125*	*125*
*p-value(F)(OLS)*	*0.583*	*0.000*	*0.319*	*0.180*	*0.187*	*0.000*	*0.0407*	*0*
*F-stat (OLS)*	*0.541*	*4.429*	*1.152*	*1.449*	*1.693*	*19.971*	*3.281*	*12.408*
*Lmin (OLS)*		*0.528 (Law)/ 0.514 (Poli Sci)*					*0.989*	

Note: The numbers in brackets are robust standard errors; * Significant at 10%, ** 5%, *** 1% in OLS, LPM or Logit regressions; + p-value equal or less than 0.10 tolerance level in backward stepwise regressions, implying that the variable ought not to be removed from the model. Lmax (Lmin) is the value of left digit ratio that maximizes (minimizes) the dependent variable, equal to -b1/(2×b2), computed only for significant values in OLS and LPM regressions.

**Table 8:** LPM and Logit Regressions of HS Honors on Left Hand 2D:4D and its Square.

*Moscow*										
	HS Honors									
	Female					Male				
Explanatory Variables	LPM	Logit	LPM	Logit	LPM		Logit	LPM	Logit	
Left Hand 2D:4D	27.599	111.982	28.410	125.207	−18.750		−88.608	−13.837	−55.615	
	*(26.915)*	*(111.303)*	*(25.405)*	*(108.830)*	*(30.665)*		*(139.901)*	*(32.019)*	*(152.586)*	
Square of Left Hand 2D:4D	−13.669	−55.465	−14.709	−64.843	9.579		45.275	6.964	27.985	
	*(13.504)*	*(55.827)*	*(12.828)*	*(54.885)*	*(15.604)*		*(71.200)*	*(16.251)*	*(77.422)*	
Left Hand 2D:4D × Faculty (Law)			−3.206* +	−13.645* +				0.089	0.558	
			*(1.681)*	*(7.326)*				*(1.918)*	*(8.481)*	
Left Hand 2D:4D × Faculty (Management)			−0.225	−0.916 +				−2.282 +	−16.901 +	
			*(2.037)*	*(8.951)*				*(1.980)*	*(12.920)*	
Left Hand 2D:4D × Faculty (Political Science)			−2.445 +	−10.384 +				−0.366	−1.741	
			*(2.127)*	*(9.016)*				*(3.188)*	*(14.859)*	
Square of Left Hand 2D:4D × Faculty (Law)			2.993* +	12.748* +				−0.183	−0.983	
			*(1.689)*	*(7.339)*				*(1.941)*	*(8.590)*	
Square of Left Hand 2D:4D × Faculty (Management)			−0.073 +	−0.329				2.072	15.728	
			*(2.032)*	*(8.932)*				*(2.018)*	*(12.994)*	
Square of Left Hand 2D:4D × Faculty (Political Science)			2.213	9.413				0.274	1.322	
			*(2.140)*	*(9.049)*				*(3.236)*	*(15.077)*	
Constant	−13.420	−56.475	−13.002	−59.522	9.449		42.382	7.243	27.100	
	*(13.403)*	*(55.444)*	*(12.595)*	*(54.023)*	*(15.059)*		*(68.689)*	*(15.780)*	*(75.184)*	
*R-squared (LPM)*	*0.003*		*0.063*		*0.001*			*0.039*		
*Adjusted R-squared (LPM)*	−*0.002*		*0.045*		−*0.005*			*0.014*		
*Pseudo R-squared (Logit)*		*0.002*		*0.046*			*0.001*		*0.036*	
*Pseudo R-squared (Stepwise)*		*0.000*		*0.042*			*0.000*		*0.025*	
*N*	*435*	*435*	*435*	*435*	*319*		*319*	*319*	*319*	
*p-value(F) (LPM, Logit)*	*0.522*	*0.535*	*0.000*	*0.001*	*0.825*		*0.814*	*0.020*	*0.203*	
*F-stat (LPM)*	*0.652*		*3.925*		*0.192*			*2.312*		
*Lmin (LPM)*			*0.536 (Law)*							

Note: The numbers in brackets are robust standard errors; * Significant at 10%, ** 5%, *** 1% in OLS, LPM or Logit regressions; + p-value equal or less than 0.10 tolerance level in backward stepwise regressions, implying that the variable ought not to be removed from the model. Lmax (Lmin) is the value of left digit ratio that maximizes (minimizes) the dependent variable, equal to -b1/(2×b2), computed only for significant values in OLS and LPM regressions.

**Table 9:** LPM and Logit Regressions of Olympiad on Left Hand 2D:4D and its Square.

*Moscow*								
	Olympiad							
	Female				Male			
Explanatory Variables	LPM	Logit	LPM	Logit	LPM	Logit	LPM	Logit
Left Hand 2D:4D	−4.014	−17.866	−7.767	−5.199	−63.072* +	−276.595** +	−50.317* +	−239.918* +
	*(24.619)*	*(114.950)*	*(23.815)*	*(126.812)*	*(32.579)*	*(139.224)*	*(29.440)*	*(140.568)*
Square of Left Hand 2D:4D	2.194	9.818	4.536 +	5.223	32.128* +	140.909** +	25.927* +	123.339* +
	*(12.407)*	*(57.832)*	*(12.047)*	*(63.895)*	*(16.569)*	*(70.792)*	*(14.915)*	*(71.263)*
Left Hand 2D:4D × Faculty (Law)			−1.020 +	−6.764 +			0.639	2.746
			*(1.666)*	*(7.623)*			*(1.737)*	*(8.095)*
Left Hand 2D:4D × Faculty (Management)			2.478 +	14.153			0.077	−0.209
			*(1.974)*	*(10.878)*			*(1.936)*	*(11.530)*
Left Hand 2D:4D × Faculty (Political Science)			2.420 +	11.038			−2.712 +	−11.970 +
			*(2.084)*	*(9.651)*			*(3.156)*	*(14.488)*
Square of Left Hand 2D:4D × Faculty (Law)			0.819	5.844			−0.930 +	−4.067 +
			*(1.674)*	*(7.623)*			*(1.757)*	*(8.196)*
Square of Left Hand 2D:4D × Faculty (Management)			−2.747 +	−15.486 +			−0.446 +	−1.665 +
			*(1.965)*	*(10.906)*			*(1.961)*	*(11.691)*
Square of Left Hand 2D:4D × Faculty (Political Science)			−2.591 +	−11.789 +			2.582	11.426
			*(2.092)*	*(9.726)*			*(3.199)*	*(14.647)*
Constant	2.115	7.178	3.685	−0.193	31.227*	134.752**	24.875*	116.502*
	*(12.206)*	*(57.088)*	*(11.793)*	*(62.968)*	*(16.006)*	*(68.403)*	*(14.530)*	*(69.311)*
*R-squared (LPM)*	*0.001*		*0.062*		*0.013*		*0.107*	
*Adjusted R-squared (LPM)*	−*0.004*		*0.045*		*0.006*		*0.084*	
*Pseudo R-squared (Logit)*		*0.001*		*0.052*		*0.010*		*0.087*
*Pseudo R-squared (Stepwise)*		*0.000*		*0.039*		*0.010*		*0.084*
*N*	*446*	*446*	*446*	*446*	*323*	*323*	*323*	*323*
*p-value(F) (LPM, Logit)*	*0.848*	*0.844*	*0.000*	*0.001*	*0.154*	*0.137*	*0.000*	*0.000*
*F-stat (LPM)*	*0.165*		*3.719*		*18850*		*5.012*	
*Lmin (LPM)*					*0.982*		*0.970 (Economics)*	

Note: The numbers in brackets are robust standard errors; * Significant at 10%, ** 5%, *** 1% in OLS, LPM or Logit regressions; + p-value equal or less than 0.10 tolerance level in backward stepwise regressions, implying that the variable ought not to be removed from the model. Lmax (Lmin) is the value of left digit ratio that maximizes (minimizes) the dependent variable, equal to -b1/(2×b2), computed only for significant values in OLS and LPM regressions.

**Table 10:** LPM and Logit Regressions of Full Scholarship on Left Hand 2D:4D and its Square.

*Moscow*								
	Full Scholarship							
	Female				Male			
Explanatory Variables	LPM	Logit	LPM	Logit	LPM	Logit	LPM	Logit
Left Hand 2D:4D	2.126	10.931	0.184	−11.150	−49.158* +	−259.535* +	−46.485* +	−277.004* +
	*(22.640)*	*(117.828)*	*(22.076)*	*(132.991)*	*(27.578)*	*(144.446)*	*(26.864)*	*(151.403)*
Square of Left Hand 2D:4D	−1.130	−5.816	0.279	7.531	25.859* +	136.198* +	24.594* +	146.418* +
	*(11.350)*	*(59.012)*	*(11.146)*	*(67.379)*	*(13.882)*	*(73.258)*	*(13.520)*	*(77.445)*
Left Hand 2D:4D × Faculty (Law)			−0.072	−1.482			0.457	2.986
			*(1.545)*	*(8.750)*			*(1.630)*	*(9.372)*
Left Hand 2D:4D × Faculty (Management)			1.885	8.420			0.189	3.741
			*(2.017)*	*(9.321)*			*(2.204)*	*(10.426)*
Left Hand 2D:4D × Faculty (Political Science)			0.699 +	2.775 +			−0.578	−4.404
			*(1.668)*	*(11.848)*			*(2.415)*	*(16.176)*
Square of Left Hand 2D:4D × Faculty (Law)			0.138	1.868			−0.442	−2.959
			*(1.544)*	*(8.807)*			*(1.639)*	*(9.553)*
Square of Left Hand 2D:4D × Faculty (Management)			−2.070 +	−9.243 +			−0.488 +	−5.083 +
			*(2.011)*	*(9.311)*			*(2.230)*	*(10.596)*
Square of Left Hand 2D:4D × Faculty (Political Science)			−0.564	−1.875			0.620	4.642
			*(1.670)*	*(11.933)*			*(2.431)*	*(16.503)*
Constant	−0.248	−4.028	0.276	4.657	23.948*	123.967*	22.590*	131.488*
	*(11.285)*	*(58.786)*	*(10.954)*	*(65.706)*	*(13.685)*	*(71.168)*	*(13.342)*	*(74.073)*
*R-squared (LPM)*	*0.000*		*0.072*		*0.022*		*0.087*	
*Adjusted R-squared (LPM)*	−*0.004*		*0.055*		*0.016*		*0.064*	
*Pseudo R-squared (Logit)*		*0.000*		*0.062*		*0.019*		*0.069*
*Pseudo R-squared (Stepwise)*		*0.000*		*0.056*		*0.019*		*0.068*
*N*	*447*	*447*	*447*	*447*	*322*	*322*	*322*	*322*
*p-value(F) (LPM, Logit)*	*0.973*	*0.972*	*0.000*	*0.000*	*0.001*	*0.008*	*0.000*	*0.001*
*F-stat (LPM)*	*0.028*		*3.893*		*6.960*		*4.833*	
*Lmin (LPM)*					*0.951*		*0.945 (Economics)*	

Note: The numbers in brackets are robust standard errors; * Significant at 10%, ** 5%, *** 1% in OLS, LPM or Logit regressions; + p-value equal or less than 0.10 tolerance level in backward stepwise regressions, implying that the variable ought not to be removed from the model. Lmax (Lmin) is the value of left digit ratio that maximizes (minimizes) the dependent variable, equal to -b1/(2×b2), computed only for significant values in OLS and LPM regressions.

Note that the results from logit regressions are similar to the LPM – dividing the logit estimates by 4 gives values that are close to the LPM estimates. The backward stepwise regressions generally confirm the results, as variables are significant whenever they are significant in the OLS/LPM/logit regressions. However, the backward stepwise regressions seem to yield more significant results - see, for instance, female and male Math Scores, male HS Honors, female Olympiads, and female Full Scholarship. This indicates that the variables are *jointly* significant, even if they are individually insignificant in the OLS/LPM/Logit regressions. In fact, note that the R-squared and F-stat are high, and the p-value(F) is low. That the variables are jointly significant is consistent with the results implied by the backward stepwise regressions which indicate that most of the variables ought to be included in the model.

It can be seen that the significant relationships are U-shaped and mostly hold for male students. Without controlling for Faculty type, there are significant U-shaped relationships between Left 2D:4D and Math Score, Olympiad and Full Scholarship for the male subsample. When we further break down the sample by Faculty type, we find significant U-shaped relationships between Left 2D:4D and the following: High School Honors for female law students; Olympiad for male economics students; Russian Score for female law and female political science students; and Full Scholarship for male economics students. Note, however, that for Russian Scores for female law and female political science students, the computed Lmin values lie outside the range of Left 2D:4D values in the Moscow sample. For law, Lmin is below the lowest 2D:4D, which indicates that the sample is on the upward-sloping part of the U-curve; while for political science, the sample is on the downward-sloping part of the U-curve (since their Lmin is above the highest 2D:4D in the sample).

Tables 11, 12, 13, and 14 present the regression results for Moscow when the Right 2D:4D is used. It can be seen that the significant quadratic relationships are inverted-U and mostly hold for females, with the exception of the High School Honors for male political science students, Olympiad for male law students and Full Scholarship for male law students. (In the latter cases, however, their respective Rmax are below the lowest 2D:4D in the sample, implying that the sample is actually on the upward-sloping part of the U-curves). That is, the U-shaped cases only hold for male samples. Without controlling for faculty type, Right 2D:4D is seen to have a significant inverted-U relationship between High School Honors for females, Olympiad for males, Math Scores of females, Russian Scores of females, and Full Scholarship for females. When considering faculty subsamples, Right 2D:4D is shown to have significant inverted-U relationships between: High School Honors for female economics and female law students: Olympiad for male law students; Math Score of female economics students; Russian Score of female economics and female political science students.

**Table 11:** OLS Regressions of Russian Score and Math Score on Right Hand 2D:4D and its Square.

*Moscow*								
	Russian Score				Math Score			
	Female		Male		Female		Male	
Explanatory Variables	OLS	OLS	OLS	OLS	OLS	OLS	OLS	OLS
Right Hand 2D:4D	1219.507*** +	1149.561* +	−358.210	−594.132	1462.162** +	1548.033*** +	−833.586	−386.7195
	*(386.220)*	*(380.681)*	*(840.250)*	*(961.873)*	*(737.697)*	*(564.843)*	*(1600.289)*	*(1253.000)*
Square of Right Hand 2D:4D	−614.585*** +	−595.052* +	176.008	304.791	−738.570** +	−771.270*** +	436.514	232.749 +
	*(191.778)*	*(190.327)*	*(425.958)*	*(494.139)*	*(366.135)*	*(279.479)*	*(819.582)*	*(646.371)*
Right Hand 2D:4D × Faculty (Law)		−57.484 +		11.199		3.351		−89.117 +
		*(35.477)*		*(50.449)*		*(53.419)*		*(72.092)*
Right Hand 2D:4D × Faculty (Management)		−52.595 +		24.576		−0.222		54.965
		*(38.187)*		*(55.692)*		*(35.739)*		*(53.825)*
Right Hand 2D:4D × Faculty (Political Science)		−83.038** +		−46.469		51.339		−27.447 +
		*(35.343)*		*(86.183)*		*(56.669)*		*(325.798)*
Square of Right Hand 2D:4D × Faculty (Law)		53.448		−15.820 +		−23.801 +		67.591
		*(35.518)*		*(51.530)*		*(52.766)*		*(72.923)*
Square of Right Hand 2D:4D × Faculty (Management)		46.244		−27.884		−9.7882 +		−66.265 +
		*(38.103)*		*(56.956)*		*(35.438)*		*(55.039)*
Square of Right Hand 2D:4D × Faculty (Political Science)		79.941** +		44.229		−73.334 +		2.309
		*(35.619)*		*(89.353)*		*(57.372)*		*(338.526)*
Constant	−522.899	−469.594**	257.593	367.324	−652.064***	−698.040**	467.597	233.046
	*(194.271)*	*(190.791)*	*(414.004)*	*(468.415)*	*(371.258)*	*(285.421)*	*(780.261)*	*(606.886)*
*R-squared (OLS)*	*0.025*	*0.114*	*0.003*	*0.046*	*0.025*	*0.503*	*0.006*	*0.449*
*Adjusted R-squared (OLS)*	*0.017*	*0.084*	−*0.008*	*0.001*	*0.011*	*0.476*	−*0.010*	*0.411*
*R-squared (Stepwise)*	*0.025*	*0.107*	*0.000*	*0.017*	*0.025*	*0.502*	*0.000*	*0.439*
*N*	*242*	*242*	*179*	*179*	*152*	*152*	*125*	*125*
*p-value(F) (OLS)*	*0.003*	*0.000*	*0.777*	*0.315*	*0.073*	*0.000*	*0.710*	*0.000*
*F-stat (OLS)*	*6.047*	*45542.000*	*0.252*	*1.178*	*2.667*	*27.740*	*0.344*	*11.880*
*Rmax (OLS)*	*0.992*	*0.976 (Econ)/ 1.035(Poli Sci)*			*0.990*	*1.004 (Economics)*		
*Rmin (LPM)*								

Note: The numbers in brackets are robust standard errors; * Significant at 10%, ** 5%, *** 1% in OLS, LPM or Logit regressions; + p-value equal or less than 0.10 tolerance level in backward stepwise regressions, implying that the variable ought not to be removed from the model. Rmax (Rmin) is the value of right digit ratio that maximizes (minimizes) the dependent variable, equal to -b1/(2×b2), computed only for significant values in OLS and LPM regressions.

**Table 12:** LPM and Logit Regressions of HS Honors on Right Hand 2D:4D and its Square.

*Moscow*								
	HS Honors							
	Female				Male			
Explanatory Variables	LPM	Logit	LPM	Logit	LPM	Logit	LPM	Logit
Right Hand 2D:4D	28.398* +	122.204	25.317* +	123.042	−1.684	−1.054	−20.747	−70.781
	*(16.380)*	*(75.279)*	*(14.748)*	*(75.679)*	*(31.134)*	*(158.239)*	*(31.622)*	*(179.175)*
Square of Right Hand 2D:4D	−13.739* +	−59.180	−12.610* +	−61.225	0.434	−1.549	10.263	34.745
	*(8.233)*	*(37.642)*	*(7.385)*	*(37.689)*	*(15.792)*	*(80.589)*	*(16.270)*	*(92.050)*
Right Hand 2D:4D × Faculty (Law)			−3.279** +	−14.069* +			1.805 +	9.223
			*(1.609)*	*(7.268)*			*(2.172)*	*(9.822)*
Right Hand 2D:4D × Faculty (Management)			−0.928 +	−4.080 +			−0.726 +	−3.227 +
			*(1.685)*	*(7.607)*			*(1.983)*	*(10.748)*
Right Hand 2D:4D × Faculty (Political Science)			−1.053 +	−4.249 +			−5.792** +	−28.893** +
			*(1.854)*	*(7.934)*			*(2.606)*	*(13.555)*
Square of Right Hand 2D:4D × Faculty (Law)			3.061* +	13.146* +			−1.918 +	−9.778
			*(1.617)*	*(7.292)*			*(2.202)*	*(10.004)*
Square of Right Hand 2D:4D × Faculty (Management)			0.623	2.804			0.488	1.899
			*(1.681)*	*(7.579)*			*(2.016)*	*(10.918)*
Square of Right Hand 2D:4D × Faculty (Political Science)			0.818	3.264			5.820** +	28.989** +
			*(1.866)*	*(7.979)*			*(2.672)*	*(13.778)*
Constant	−14.141***	−62.948	−12.003	−60.940	1.520	1.611	2405398.000	35.468
	*(8.144)*	*(37.620)*	*(7.405)*	*(38.125)*	*(15.337)*	*(77.635)*	*(15.390)*	*(87.225)*
*R-squared (LPM)*	*0.010*		*0.070*		*0.004*		*0.063*	
*Adjusted R-squared (LPM)*	*0.005*		*0.052*		−*0.003*		*0.038*	
*Pseudo R-squared (Logit)*		*0.007*		*0.052*		*0.003*		*0.054*
*Pseudo R-squared (Stepwise)*		*0.000*		*0.047*		*0.000*		*0.038*
*N*	*435*	*435*	*435*	*435*	*319*	*319*	*319*	*319*
*p-value(F) (LPM, Logit)*	*0.047*	*0.085*	*0.000*	*0.000*	*0.554*	*0.558*	*0.004*	*0.016*
*F-stat (LPM)*	*3.071*		*5.117*		*0.592*		*2.913*	
*Rmax (LPM)*	*1.034*		*1.004(Econ)/ 1.154(Law)*					
*Rmin (LPM)*							*0.498 (Poli Sci)*	

Note: The numbers in brackets are robust standard errors; * Significant at 10%, ** 5%, *** 1% in OLS, LPM or Logit regressions; + p-value equal or less than 0.10 tolerance level in backward stepwise regressions, implying that the variable ought not to be removed from the model. Rmax (Rmin) is the value of right digit ratio that maximizes (minimizes) the dependent variable, equal to -b1/(2×b2), computed only for significant values in OLS and LPM regressions.

**Table 13:** LPM and Logit Regressions of Olympiad on Right Hand 2D:4D and its Square.

*Moscow*								
	Olympiad							
	Female				Male			
Explanatory Variables	LPM	Logit	LPM	Logit	LPM	Logit	LPM	Logit
Right Hand 2D:4D	−0.175	−0.7937	−0.955	31.4653	50.850* +	266.6381*** +	16.612 +	157.5927 +
	*(17.223)*	*(82.3275)*	*(14.164)*	*(86.8018)*	*(25.999)*	*(152.6926)*	*(25.964)*	*(180.0398)*
Square of Right Hand 2D:4D	0.122	0.5637	0.640	−14.9175	−25.542* +	−133.9596*** +	−7.014	−74.4144
	*(8.601)*	(41.0894)	*(7.109)*	(43.1791)	*(13.210)*	(77.4587)	*(13.369)*	*(92.1579)*
Right Hand 2D:4D × Faculty (Law)			−2.073 +	−11.1257 +			3.421* +	16.2373*** +
			*(1.584)*	*(7.1597)*			*(1.922)*	*(9.7922)*
Right Hand 2D:4D × Faculty (Management)			−0.790 +	−5.1301 +			0.916 +	−3.1928 +
			*(1.528)*	*(7.6444)*			*(1.772)*	*(10.6712)*
Right Hand 2D:4D × Faculty (Political Science)			2.576 +	14.5085			−1.593 +	−10.1153 +
			*(1.790)*	*(9.9112)*			*(2.704)*	*(13.6169)*
Square of Right Hand 2D:4D × Faculty (Law)			1.872 +	10.1934 +			−3.768* +	−17.8090*** +
			*(1.595)*	*(7.1613)*			*(1.950)*	*(9.9671)*
Square of Right Hand 2D:4D × Faculty (Management)			0.512	3.7685			−1.318	1.2536
			*(1.531)*	*(7.5965)*			*(1.812)*	*(10.8045)*
Square of Right Hand 2D:4D × Faculty (Political Science)			−2.754 +	−15.3794 +			1.438	9.4827
			*(1.798)*	*(10.0577)*			*(2.767)*	*(13.8278)*
Constant	0.349	−0.6357	0.771	−16.6962	−24.969***	−133.3367***	−9.031	−82.8869
	*(8.618)*	*(41.2198)*	*(7.103)*	*(43.7047)*	*(12.784)*	*(75.2157)*	*(12.631)*	*(87.9565)*
*R-squared (LPM)*	*0.000*		*0.065*		*0.008*		*0.118*	
*Adjusted R-squared (LPM)*	−*0.005*		*0.048*		*0.002*		*0.095*	
*Pseudo R-squared (Logit)*		*0*		*0.0544*		*0.0072*		*0.0972*
*Pseudo R-squared (Stepwise)*								
*N*	*446*	*446*	*446*	*446*	*323*	*323*	*323*	*323*
*p-value(F) (LPM, Logit)*	*0.992*	*0.9923*	*0.000*	*0.0007*	*0.110*	*0.1757*	*0.000*	*0*
*F-stat (LPM)*	*0.008*		*3.921*		*2.219*		*6.090*	
*Rmax (LPM)*					*0.995*		*0.454 (Law)*	
*Rmin (LPM)*								

Note: The numbers in brackets are robust standard errors; * Significant at 10%, ** 5%, *** 1% in OLS, LPM or Logit regressions; + p-value equal or less than 0.10 tolerance level in backward stepwise regressions, implying that the variable ought not to be removed from the model. Rmax (Rmin) is the value of right digit ratio that maximizes (minimizes) the dependent variable, equal to -b1/(2×b2), computed only for significant values in OLS and LPM regressions.

**Table 14:** LPM and Logit Regressions of Full Scholarship on Right Hand 2D:4D and its Square.

*Moscow*								
	Full Scholarship							
	Female				Male			
Explanatory Variables	LPM	Logit	LPM	Logit	LPM	Logit	LPM	Logit
Right Hand 2D:4D	30.968* +	145.569* +	27.158*	147.098*	−13.4755	−82.8777	−40.81	−250.6419
	*(18.322)*	*(80.421)*	*(16.375)*	*(84.711)*	*(31.359)*	*(152.694)*	*(31.540)*	*(169.683)*
Square of Right Hand 2D:4D	−15.412* +	−72.427* +	−13.358	−72.359***	7.666 +	45.855 +	22.528 +	137.110 +
	*(9.160)*	*(40.195)*	*(8.218)*	*(42.343)*	*(15.814)*	*(77.514)*	*(16.132)*	*(88.239)*
Right Hand 2D:4D × Faculty (Law)			0.279	1.397			3.523* +	19.050* +
			*(1.495)*	*(7.439)*			*(1.875)*	*(10.964)*
Right Hand 2D:4D × Faculty (Management)			−1.835 +	−7.719 +			1.157	9.603
			*(1.647)*	*(7.553)*			*(2.272)*	*(11.762)*
Right Hand 2D:4D × Faculty (Political Science)			1.118 +	6.961 +			−0.421	−3.889
			*(1.554)*	*(8.903)*			*(2.552)*	*(15.270)*
Square of Right Hand 2D:4D × Faculty (Law)			−0.219	−1.056			−3.576* +	−19.414* +
			*(1.499)*	*(7.470)*			*(1.903)*	*(11.241)*
Square of Right Hand 2D:4D × Faculty (Management)			1.635	6.824			−1.505 +	−11.232 +
			*(1.642)*	*(7.533)*			*(2.305)*	*(12.040)*
Square of Right Hand 2D:4D × Faculty (Political Science)			−0.990	−6.109			0.459	4.141
			*(1.563)*	*(8.926)*			*(2.586)*	*(15.713)*
Constant	−14.785	−71.939***	−13.041	−73.586***	6.474	37.712	19.032	114.712
	*(9.158)*	*(40.200)*	*(8.189)*	*(42.485)*	*(15.535)*	*(75.153)*	*(15.438)*	*(81.717)*
*R-squared (LPM)*	*0.008*		*0.079*		*0.014*		*0.095*	
*Adjusted R-squared (LPM)*	*0.003*		*0.062*		*0.007*		*0.071*	
*Pseudo R-squared (Logit)*		*0.006*		*0.067*		*0.011*		*0.074*
*R-squared (Stepwise)*		*0.006*		*0.053*		*0.010*		*0.068*
*N*	*447*	*447*	*447*	*447*	*322*	*322*	*322*	*322*
*p-value(F) (LPM, Logit)*	*0.235*	*0.190*	*0.000*	*0.000*	*0.062*	*0.098*	*0.000*	*0.001*
*F-stat (LPM)*	*1.454*		*4.806*		*2.806*		*4.648*	
*Rmax (LPM)*	*1.005*							
*Rmin (LPM)*							*0.493 (Law)*	

Note: The numbers in brackets are robust standard errors; * Significant at 10%, ** 5%, *** 1% in OLS, LPM or Logit regressions; + p-value equal or less than 0.10 tolerance level in backward stepwise regressions, implying that the variable ought not to be removed from the model. Rmax (Rmin) is the value of right digit ratio that maximizes (minimizes) the dependent variable, equal to -b1/(2×b2), computed only for significant values in OLS and LPM regressions.

Table 15 reports regression results for Manila using Left 2D:4D. We find that the only significant result is for females–their Mathematics Weighted Average and Left 2D:4D have an inverted-U relationship. Table 16 reports results using Right 2D:4D, where it can be seen that Right 2D:4D is significantly related to both Economics and Mathematics Weighted Average in a U-shaped fashion, both for males and females. (Backward stepwise regressions yield significant results only for the Right 2D:4D, and mostly for females).

**Table 15:** OLS Regressions of Economics Weighted Average and Mathematics Weighted Average on Left Hand 2D:4D and its Square.

*Manila*								
	Economics Weighted Average				Mathematics Weighted Average			
	Female		Male		Female		Male	
Explanatory Variables	OLS		OLS		OLS		OLS	
Left hand 2D:4D	1.175	5.067	3.127	141.498	1.076	106.71 ***	5.865	25.084
	*(2.388)*	*(79.256)*	*(3.104)*	*(123.920)*	*(2.636)*	*(61.120)*	*(4.150)*	*(154.448)*
Square of Left hand 2D:4D		−2.015		−72.476		−54.615***		−10.066
		*(41.387)*		*(65.350)*		*(32.189)*		*(80.880)*
Constant	1.273	−0.604	−0.254	−72.476	1.242	−49.774	−2.970	−12.134
	*(2.313)*	*(37.942)*	*(2.968)*	*(65.350)*	*(2.556)*	*(28.994)*	*(3.968)*	*(73.705)*
*R-squared (OLS)*	*0.0035*	*0.0035*	*0.0211*	*0.042*	*0.0023*	*0.0194*	*0.0408*	*0.041*
*Adjusted R-squared (OLS)*	−*0.0108*	−*0.0254*	*0.0003*	*0.0004*	−*0.0115*	−*0.0083*	*0.0204*	−*0.0007*
*R-squared (Stepwise)*	*0.000*	*0.000*	*0.000*	*0.000*	*0.000*	*0.000*	*0.000*	*0.000*
*N*	*72*	*72*	*49*	*49*	*74*	*74*	*49*	*49*
*p-value(F) (OLS)*	*0.624*	*0.8867*	*0.3191*	*0.3725*	*0.6842*	*0.4996*	*0.1641*	*0.3818*
*F-stat (OLS)*	*0.24*	*0.12*	*1.01*	*1.01*	*0.17*	*0.7*	*2*	*0.98*
*Lmax (OLS)*						0.977		

Note: The numbers in brackets are OLS standard errors; *Significant at 10%, ** 5%, *** 1%; + p-value equal or less than 0.10 tolerance level in backward stepwise regressions, implying that the variable ought not to be removed from the model. Lmax (Lmin) is the value of left digit ratio that maximizes (minimizes) the dependent variable, equal to -b1/(2×b2), computed only for significant values.

**Table 16:** OLS Regressions of Economics Weighted Average and Mathematics Weighted Average on Right Hand 2D:4D and its Square.

*Manila*								
	Economics Weighted Average				Mathematics Weighted Average			
	Female		Male		Female		Male	
Explanatory Variables	OLS		OLS		OLS		OLS	
Right hand 2D:4D	2.614	−113.175*** +	3.450	−177.03* +	4.3406*	−94.946***	0.484	−197.373***
	*(2.081)*	*(63.250)*	*(2.723)*	*(58.930)*	*(2.329)*	*(52.119)*	*(3.739)*	*(113.408)*
Square of Right hand 2D:4D		57.595*** +		94.147* +		49.386*** +		103.215***
		*(30.789)*		*(30.960)*		*(25.662)*		*(60.004)*
Constant	−0.193	57.595	−0.614	85.755	−2.038	47.793	2.166	96.854
	*(2.074)*	*(32.439)*	*(2.645)*	*(27.992)*	*(2.321)*	*(26.432)*	*(3.631)*	*(53.499)*
*R-squared (OLS)*	0.022	0.063	*0.033*	0.0996	0.046	0.0685	*0.0004*	0.0442
*Adjusted R-squared (OLS)*	*0.008*	*0.0353*	*0.0124*	*0.0604*	*0.0328*	*0.0423*	−*0.0209*	*0.0027*
*R-squared (Stepwise)*	*0.000*	*0.063*	*0.000*	*0.0996*	*0.048*	*0.048*	*0.000*	*0.000*
*N*	72	72	*49*	*49*	*74*	74	*49*	49
*p-value(F) (OLS)*	*0.2131*	*0.1078*	*0.2114*	*0.0896*	*0.0665*	*0.0805*	*0.8976*	*0.3533*
*F-stat (OLS)*	*1.58*	*2.3*	*1.61*	*2.54*	*3.47*	*2.61*	*0.02*	*1.06*
*Rmin (OLS)*		*0.983*		*0.94*		*0.961*		*0.956*

Note: The numbers in brackets are OLS standard errors; * Significant at 10%, ** 5%, *** 1%; + p-value equal or less than 0.10 tolerance level in backward stepwise regressions, implying that the variable ought not to be removed from the model. Rmax (Rmin) is the value of right digit ratio that maximizes (minimizes) the dependent variable, equal to -b1/(2×b2), computed only for significant values.

All these results indicate the following patterns across Manila and Moscow. In Moscow, using the right (left) hand generates inverted-U (U-shaped) curves while in Manila, using the left (right) hand generates the inverted-U (U-shape).That is, without accounting for gender, the results for Manila are opposite of those for Moscow depending on which hand is used. However, when we consider gender subsamples, both Manila and Moscow seem to produce a consistent trend in that the U-shaped curve seems to be more associated with male students. In Manila, while Right 2D:4D also generates U-shaped curves for females, note that the only significant results for males are U-shaped. In Moscow, it seems that irrespective of which hand is used, the significant results for males are almost always U-shaped.

### Conclusion

We have shown in both Moscow and Manila that the degree to which prenatal testosterone is linked to academic achievement exhibits some nonlinearity, and the precise relationship is dependent on gender, faculty, or subject choice, and on which hand is used to proxy for prenatal testosterone.

To the extent we do not yet understand the precise mechanism through which prenatal androgens manifest themselves in the right versus the left hand, this suggests that much more needs to be done to learn how we can use these measures to study the effects of prenatal testosterone on achievement. Our research combined with the findings of [17] make clear that the potential nonlinearity in prenatal testosterone’s effects coupled to the differential benefits of abstract reasoning in different contexts would lead to highly particular links of 2D4D to achievement depending on field or choice of achievement measure. We might speculate for example that the strong results in sports or in financial trading are in areas where there is no tradeoff to greater abstract reasoning combined with greater risk taking. In other situations, nonlinearity is more likely to emerge and it might be harder to discern these interactions without further identifying restrictions.
