# Correction: 2D:4D Asymmetry and Gender Differences in Academic Performance

**DOI:** 10.1371/annotation/8e05e33b-58cf-4983-b021-3a65d27ef2a1

**Published:** 2013-03-26

**Authors:** John V. C. Nye, Gregory Androuschak, Desirée Desierto, Garett Jones, Maria Yudkevich

The first author John V. C. Nye is missing an affiliation. He is affiliated with numbers 1 and 2: the Department of Economics, George Mason University, Fairfax, Virginia, United States of America and the Laboratory for Institutional Analysis of Economic Reforms, National Research University “Higher School of Economics”, Moscow, Russia.

